# A mathematical model relates intracellular TLR4 oscillations to sepsis progression

**DOI:** 10.1186/s13104-018-3561-9

**Published:** 2018-07-11

**Authors:** Razvan C. Stan, Francisco G. Soriano, Maristela M. de Camargo

**Affiliations:** 1 Institute of Biomedical Sciences, University of São Paulo, São Paulo, CEP 05508-900 Brazil; 2 University Hospital, University of Sao Paulo, São Paulo, CEP 05508-000 Brazil

**Keywords:** Sepsis, Inflammation, Intracellular trafficking, Ordinary differential equations, Oscillations

## Abstract

**Objective:**

Oscillations of physiological parameters describe many biological processes and their modulation is determinant for various pathologies. In sepsis, toll-like receptor 4 (TLR4) is a key sensor for signaling the presence of Gram-negative bacteria. Its intracellular trafficking rates shift the equilibrium between the pro- and anti-inflammatory downstream signaling cascades, leading to either the physiological resolution of the bacterial stimulation or to sepsis. This study aimed to evaluate the effects of TLR4 increased expression and intracellular trafficking on the course and outcome of sepsis.

**Results:**

Using a set of three differential equations, we defined the TLR4 fluxes between relevant cell organelles. We obtained three different regions in the phase space: (1) a limit-cycle describing unstimulated physiological oscillations, (2) a fixed-point attractor resulting from moderate LPS stimulation that is resolved and (3) a double-attractor resulting from sustained LPS stimulation that leads to sepsis. We used this model to describe available hospital data of sepsis patients and we correctly characterize the clinical outcome of these patients.

**Electronic supplementary material:**

The online version of this article (10.1186/s13104-018-3561-9) contains supplementary material, which is available to authorized users.

## Introduction

The immune system is replete with oscillations of various parameters needed for mounting an appropriate response upon stimulation. TLR4 is an important bacterial recognition that serves as a link between innate and adaptive immunity [1]. In particular, TLR4 experiences a significant upregulation in mRNA production and presentation to the cell surface at the initial stages of sepsis in both humans and experimental models [2]. An emerging theme in TLR4 signaling posits that its cellular localization is determinant for its functions [3]. Throughout the continuum of sepsis, complete TLR4 signaling includes not only the initial surface-bound pro-inflammatory signaling, but also its subsequent endocytosis and intracellular trafficking. This results in competing endosomal anti-inflammatory cytokine production and further into either receptor recycling to cell membrane or signal termination within endolysosomes. Initial responsiveness to LPS is therefore regulated by the concentration of cell surface TLR4 that depends in turn on TLR4 expression, on TLR4 trafficking from the Golgi apparatus to the plasma membrane and on the amount of TLR4 already internalized in endosomes [4].

## Main text

### Mechanistic model of TLR4 trafficking

**Fig. 1:**
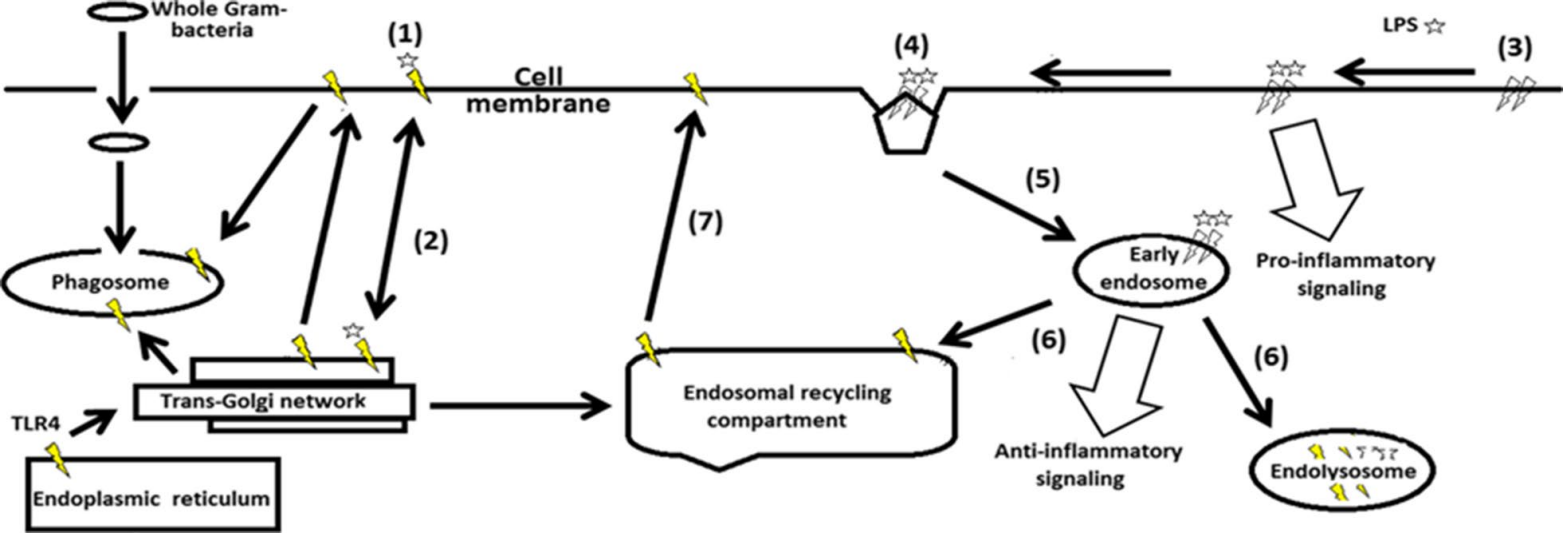
TLR4 distribution and activation between different cell compartments. Star symbols represent single LPS ligands; yellow thunder symbols depict single TLR4, white thunders describe signaling-competent TLR4 dimers. Numbers indicate the steps of LPS binding, followed by TLR4 trafficking and signaling events

An overview of the known TLR4 intracellular trafficking routes that influence its signaling is presented in Fig. 1.

Upon endotoxin stimulation, initial TLR4 immobilization (step 1) may lead to monomeric LPS being internalized and trafficked to the Golgi apparatus within seconds of stimulation, without activating TLR4 (step 2). This is followed by TLR4 clustering (step 3) with monomeric LPS [5]. Internalization by either clathrin- and dynamin-mediated processes [6] results in a switch in TLR4 signaling pathways by means of different adaptors (step 4). Provided that TLR4 endocytosis has occurred, signaling continues induction of type-I interferons [7] from early endosomes (step 5). For the signal to be terminated (step 6), the TLR4 complex is ubiquitinated and marked for lysosomal degradation. Alternatively, TLR4 can be recycled for new signaling cycles back to the cell surface via the endosomal recycling compartment (step 7). Under limit-cycle unstimulated physiological oscillations, cell surface TLR4 are present in low concentrations in macrophages or are undetectable in dendritic cells, with most resident TLR4 being distributed in the Golgi apparatus [8]. Rapid TLR4 mobilization to cell membrane follows LPS activation [9], canceling the downregulation present in physiological conditions that serves to desensitize cells to low endotoxin levels. While the overall sequence of TLR4 activation has been elucidated, the rates of TLR4 trafficking are not quantified, nor are available absolute numbers for TLR4 expression on cell surfaces.

### Simulation of TLR4 trafficking routes

In order to simulate in silico the initial TLR4 trafficking events between the endosomal recycling compartment (ERC) and the trans-Golgi network (TGN) to and from cell surface and within the early endosomes–endolysosome (EE) system, we have constructed a dynamic model (Additional file 1), based on the three ordinary differential equations presented below:

1 $$ x^{\prime}[t] = \varphi x[t] - y[t]z[t] $$

2 $$ y^{\prime}\left[ t \right] = x\left[ t \right] - \beta y\left[ t \right] - \alpha y[t] $$

3 $$ z^{\prime}[t] = x[t]y[t] - z[t]\left( {\gamma - \sigma } \right) $$

where: x = concentration of TLR4 in TGN and ERC, y = concentration of TLR4 in endosomes/endolysosomes (EE), z = concentration of TLR4 on cell surface, ϕ = rate of TLR4 mRNA production, β = rate of TLR4 trafficked to lysosomes from endosomes, α = rate of TLR4 retroactively trafficked to ERC from endosomes, γ = rate of TLR4 on cell surface trafficked to TGN, σ = rate of TLR4 on cell surface trafficked to endosomal system.

The TLR4 flux in the system as indicated by Eq. (1) is influenced by the TRAM distribution within ERC that shifts onto the enlarged CD14/LPS-positive endosomes upon TLR4 activation [10]. The adaptor TRAM is also constitutively present at the plasma membrane anchored at a N-terminal myristoylation site and traffics concomitantly the TLR4 signaling complex unidirectionaly to the endosomal system [11]. This synergy allows for the anti-inflammatory signaling phase to take preponderance, possibly due to unique TLR4 conformation brought on by the endosomal acidic environment, as previously proposed [12]. These events are dominant after about 30 min upon LPS stimulation [6], allowing for TLR4 to traffic, in a first stage mostly bidirectionally from the ERC to EE (Eq. 2). The small GTPase Rab7b is upregulated upon LPS exposure in the early endosomes and is a key regulator of intracellular trafficking of the TLR4 signaling complex to either late endosomes/lysosomes for signal termination (parameter β), or to ERC (parameter α) [13]. In Rab7b-silenced macrophages, after LPS stimulation, continued TLR4 presence only in the EE system has adverse effects as to its prolonged anti-inflammatory signaling [14]. Equation (3) describes the TLR4 cell surface concentration changes as the difference between the pool of available TLR4 in TGN + ERC and in EE, and the TLR4 that is actively being prevented from clustering on the cell surface (so as to increase downstream signaling), be it directly from the surface towards TGN (parameter γ) or towards EE (parameter σ).

### Results and discussion

**Fig. 2:**
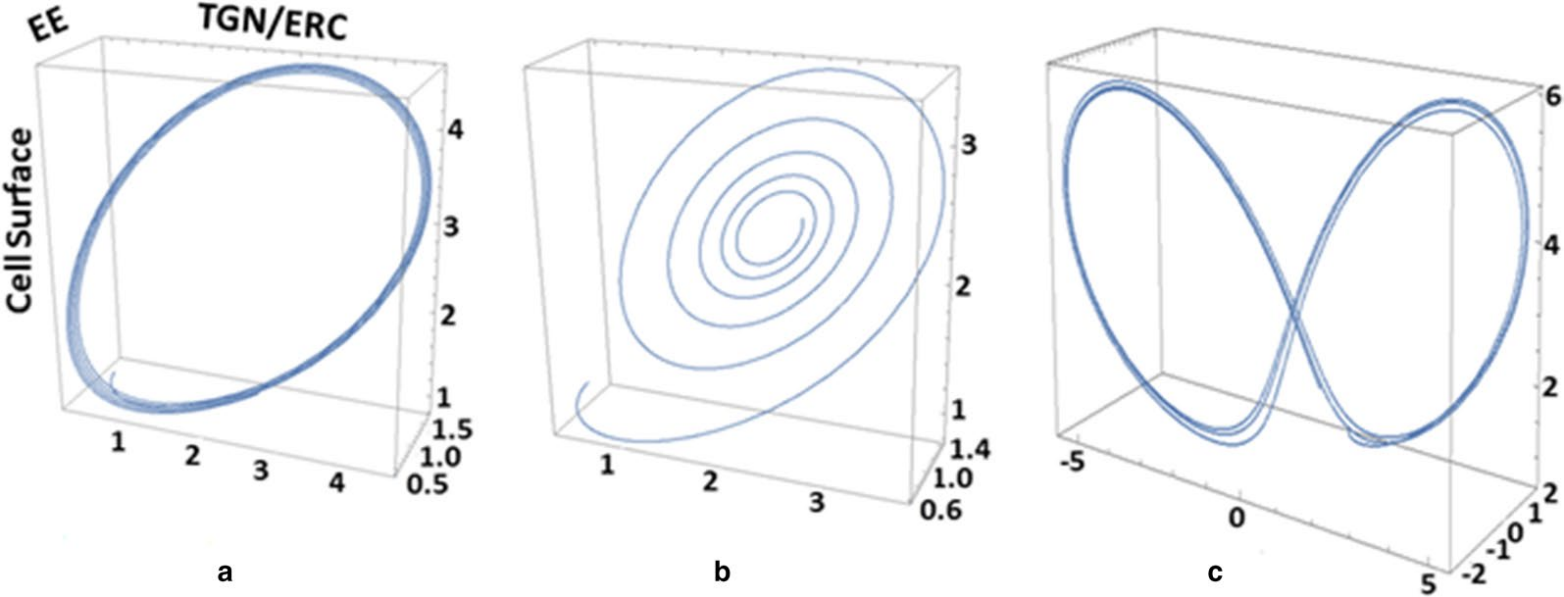
Simulated TLR4 cellular distribution during sepsis. **a** Attractive limit cycle representing steady-state oscillations. **b** Fixed-point attractor obtained following a low to medium (ϕ < 1.2) *tlr4* mRNA increase that temporarily augments TLR4 concentrations on the cell surface and thereafter within the EE system. **c** Double-attractor obtained upon increasing *tlr4* mRNA, that leads to high TLR4 concentrations oscillating indeterminately between EE and cell membrane. X axis = TLR4 concentration in TGN/ERC. Y axis = concentration of TLR4 in EE. Z axis = concentration of TLR4 on cell surface. Units represent fold changes

In the absence of available data from the literature, the parameter values for ϕ, β, γ, σ and ϕ were varied until a stable limit cycle was obtained, a characteristic of protein expression, as previously shown [15]. The first four parameters were kept constant to reflect the steady-state, non-stimulated oscillations in the TLR4 intracellular trafficking, while the ϕ-parameter that was determined experimentally elsewhere [16] was allowed to vary. Using Eqs. 1–3, we sought to model the cellular regimes that are impacted by the overall TLR4 sensitivity to LPS, as reflected by the initial rate of *tlr4* mRNA synthesis, upon sepsis diagnosis and prior to clinical intervention. The ϕ-parameter augments markedly in experimental models of sepsis and directly correlates with mortality, with peak increases between 1 and 3 h post sepsis induction [2]. As such, its oscillations will determine TLR4 changes within various relevant cell compartments and dictate the timing and preponderance of the pro- and anti-inflammatory responses. We defined three regions in the phase space for the plasma membrane and intracellular TLR4 distribution, based on the variations of ϕ-parameter (Additional file 2): (i) a steady-state with TLR4 expression and concentration oscillating within a narrow margin throughout the relevant cell compartments, (ii) a low to medium *tlr4* mRNA production following LPS stimulation that results in an initial increase of TLR4 concentration on the cell surface and subsequently in the endosomal system, followed by a regulated decrease, (iii) a third, high *tlr4* mRNA output matching increasing LPS stimulation where TLR4 concentrations oscillate stably and irreversibly on the cell surface and within the EE. The variations in *tlr4* mRNA measured in the patients served as the first parameter (ϕ) to be changed, responsible for initial TLR4 distribution within the relevant cell compartments. TLR4 is unique among other pathogen-recognition receptors in that its intracellular trafficking is determinant for the inflammatory signaling it initiates [3]. Depending on initial conditions and rate changes, the ensuing orbits either approach stable fixed points or undergo variations, each having a different physiological interpretation, as presented in Fig. 2.

#### a. Physiological variations in TLR4 concentrations

In all simulations, we assumed that initial expression levels on the cell surface, TGN/ERC and EE are low. For steady-state conditions, we proposed that TLR4 concentration oscillations are of low amplitude, reflecting the experimental data on *tlr4* mRNA in human monocytes in vitro [17]. A stable limit cycle is achieved with ϕ = 1.2, β = 3.6, α = 1.2, γ = 2.4, σ = 1.3 (Fig. 2a).

#### b. Sepsis progression and resolution

We propose that following a moderate LPS stimulation, TLR4 levels initially increase in order to proportionally signal the Gram-negative bacterial presence [8]. A fixed-point attractor is obtained with ϕ < 1.2, β = 3.6, α = 1.2, γ = 2.4, σ = 1.3 (Fig. 2b).

#### c. Sepsis progression and mortality

Upon increasing LPS stimulation, we assumed that *tlr4* mRNA rates are amplified proportionally, the result of which leads to the system moving to a double-attractor. TLR4 concentrations oscillate with highest amplitude and indefinitely between cell surface and EE compartments, with no signal resolution, using ϕ > 1.2, β = 3.6, α = 1.2, γ = 2.4, σ = 1.3 (Fig. 2c).

We note that this model, when using the initial, pre-treatment rates of *tlr4* mRNA from the patient cohort yielded appropriate descriptions of both the clinical outcome in 8 out of 10 patients [16], and the category of attractor each patient belongs to (Additional file 2). Patients whose TLR4 concentrations changes evolved towards one attractor survived sepsis (patients #1, 4, 5, and 8). In contrast, those patients that presented a double-attractor state for TLR4 died within 3 days after ICU admission (patients #3, 6, 7, and 10). As a test to the sensitivity and specificity of our model, patient #2 died 9 days after ICU admittance due to *Candida albicans* infection, a pathogen known to stimulate both TLR2 and TLR4 and is commonly associated with severe immunosuppression [18]. Patient #9 survived with negative microbiological cultures, a result of false-negative cultures or sepsis without infection, a situation not accounted for in our model. We have used initial *tlr4* mRNA expression levels from sepsis patients in a dynamic model in order to describe the distribution of TLR4 within the cell surface compartment (pro-inflammatory role), or intracellularly (anti-inflammatory and signal termination functions). We discriminated Gram-negative infections from the overall cohort and correctly described the clinical outcome of 8/10 patients. Confirming this model with in vivo measurements of TLR4 intracellular trafficking rates would provide further insight into their contribution to sepsis onset and progression.

## Limitation

The study was unable to account for phagosome signaling of whole Gram negative bacteria, nor for the additional TLR4 subpopulation that trafficks from ERC to phagosome after LPS stimulation.

## Additional files

**Additional file 1.** Mathematica code.

**Additional file 2: Table S1.** Modeling and clinical data of sepsis patients. **Table S2.** Clinical qPCR data.

## Abbreviations

TLR: toll-like receptor
ERC: endosomal recycling compartment
TGN: trans-Golgi network
EE: endosomes–endolysosome
