# Supplementary material for: A mathematical model relates intracellular TLR4 oscillations to sepsis progression
Source: BMC Res Notes. 2018 Jul 11;11:462. doi: 10.1186/s13104-018-3561-9 (PMC6042260; doi:10.1186/s13104-018-3561-9)
Supplement: Supplementary file 1 — Additional file 1. Mathematica code. [file 13104_2018_3561_MOESM1_ESM.docx]

A mathematical model relates intracellular TLR4 oscillations to sepsis progression

Authors: Razvan C. Stan^a*^, Francisco G. Soriano^b^, Maristela M. de Camargo^a*^

^a^Institute of Biomedical Sciences, University of São Paulo, CEP 05508-900, São Paulo, Brazil, [strazvan@usp.br](mailto:strazvan@usp.br); [mmcamar@usp.br](mailto:mmcamar@usp.br)

^b^University Hospital, University of Sao Paulo, CEP 05508-000, São Paulo, Brazil, [gsoriano@usp.br](file:///C:\\Users\\s_raz\\Desktop\\Septic%20articles\\BMC\\gsoriano@usp.br)

*Corresponding author: [strazvan@usp.br](mailto:strazvan@usp.br) (R.C.S.)

**Mathematical model and source code**

The dynamic system was constructed with Mathematica 10 (Wolfram Research, USA) using three ordinary differential equations, in order to describe TLR4 trafficking between different cell compartments. Units represent fold changes. The simulation package can be downloaded from <https://dataverse.harvard.edu/dataset.xhtml?persistentId=doi:10.7910/DVN/NRLZRK> and run using the CDF player from Wolfram Research.

Manipulate[Clear[x, y, z, t]; Module[{soln$}, With[{\[Phi]$ = \[Phi]\[Phi], \[Gamma]$ = \[Gamma]\[Gamma], \[Beta]$ = \[Beta]\[Beta], \[Alpha]$ = \[Alpha]\[Alpha], \[Sigma]$ = \[Sigma]\[Sigma], x0$ = xx0, y0$ = yy0, z0$ = zz0},

soln$ = Quiet[NDSolve[SetPrecision[{Derivative[1][x][t] == \[Phi]$*x[t] - y[t]*z[t], Derivative[1][y][t] == x[t] - y[t]*(\[Beta]$) - y[t]*(\[Alpha]$),

Derivative[1][z][t] == x[t]*y[t] - z[t]*(\[Gamma]$ - \[Sigma]$), x[0] == x0$, y[0] == y0$, z[0] == z0$}, Infinity], {x[t], y[t], z[t]}, {t, 0, tt},

PrecisionGoal -> ControlActive[2, 8], WorkingPrecision -> ControlActive[MachinePrecision, 20]]];

ParametricPlot3D[Evaluate[{x[t], y[t], z[t]} /. soln$], {t, 0, soln$[[1,1,2,0,1,1,2]]}, PlotPoints -> ControlActive[100, 200],

MaxRecursion -> ControlActive[4, 6], Axes -> True, Boxed -> True, AxesLabel -> {TLR4 - Golgi/ERC, TLR4 - Endosomes, TLR4 - Surface},

ImageSize -> {400, 400}, PlotRange -> All]]], {{tt, 1, "Time"}, 1, 72, 3, ImageSize -> Small, Appearance -> "Labeled"}, Delimiter,

Style["Parameters", Bold], {{\[Alpha]\[Alpha], 1.2, "\[Alpha]"}, 0, 10, 0.1, ImageSize -> Small, Appearance -> "Labeled"},

{{\[Gamma]\[Gamma], 2.4, "\[Gamma]"}, 0, 10, 0.1, ImageSize -> Small, Appearance -> "Labeled"}, {{\[Beta]\[Beta], 3.6000000000000005, "\[Beta]"}, 0, 10, 0.1, ImageSize -> Small,

Appearance -> "Labeled"}, {{\[Sigma]\[Sigma], 1.1999999999999997, "\[Sigma]"}, 0, 10, 0.1, ImageSize -> Small, Appearance -> "Labeled"},

{{\[Phi]\[Phi], 1.4000000000000001, "\[Phi] "}, 0, 10, 0.1, ImageSize -> Small, Appearance -> "Labeled"}, Delimiter, Style["Initial Conditions", Bold],

{{xx0, 2, "\!\(\*SubscriptBox[\(x\), \(0\)]\)"}, 0, 10, 1, ImageSize -> Small, Appearance -> "Labeled"},

{{yy0, 2, "\!\(\*SubscriptBox[\(y\), \(0\)]\)"}, 0, 10, 0.25, ImageSize -> Small, Appearance -> "Labeled"},

{{zz0, 2, "\!\(\*SubscriptBox[\(z\), \(0\)]\)"}, 0, 10, 1, ImageSize -> Small, Appearance -> "Labeled"}, ControlPlacement -> Right,

AutorunSequencing -> {3, 4, 5}, TrackedSymbols -> Manipulate]
