# Supplementary material for: A mathematical model relates intracellular TLR4 oscillations to sepsis progression
Source: BMC Res Notes. 2018 Jul 11;11:462. doi: 10.1186/s13104-018-3561-9 (PMC6042260; doi:10.1186/s13104-018-3561-9)
Supplement: Supplementary file 2 — Additional file 2: Table S1. Modeling and clinical data of sepsis patients. Table S2. Clinical qPCR data. [file 13104_2018_3561_MOESM2_ESM.docx]

A mathematical model relates intracellular TLR4 oscillations to sepsis progression

Authors: Razvan C. Stan^a*^, Francisco G. Soriano^b^, Maristela M. de Camargo^a*^

^a^Institute of Biomedical Sciences, University of São Paulo, CEP 05508-900, São Paulo, Brazil, [strazvan@usp.br](mailto:strazvan@usp.br); [mmcamar@usp.br](mailto:mmcamar@usp.br)

^b^University Hospital, University of Sao Paulo, CEP 05508-000, São Paulo, Brazil, [gsoriano@usp.br](file:///C:\Users\s_raz\Desktop\Septic%20articles\BMC\gsoriano@usp.br)

*Corresponding author: [strazvan@usp.br](mailto:strazvan@usp.br) (R.C.S.)

**Patient gene expression data**

The model was tested using mRNA data published elsewhere, part of a larger, separate investigation into daily (days 1-5) expression of *tlr4* and *grp78* (glucose regulated protein 78 kDa) mRNA in sepsis patients [1]. Briefly, blood samples were collected every 24 hours (up to 5 days) from clinical and surgical adult patients diagnosed with sepsis within the first 24 hours of hospital admission and referred to the Intensive Care Unit of the University Hospital at the University of São Paulo. Supplemental Table 1 presents a clinical overview of the patients.

**Additional file 2: Table S1.** Modeling and clinical data for the patients used in this study

| Patient # | 1 | 2 | 3 | 4 | 5 | 6 | 7 | 8 | 9 | 10 |
| --- | --- | --- | --- | --- | --- | --- | --- | --- | --- | --- |
| Data points | 5 | 4 | 5 | 5 | 2 | 6 | 2 | 6 | 6 | 3 |
| Attractor | 1 | 1 | 2 | 1 | 1 | 2 | 2+ | 1 | 2 | 2 |
| Day of death/release | 6 | 9 | 28 | 10 | 22 | 6 | 2 | 20 | 10 | 3 |
| Outcome | Discharge | Death | Death | Discharge | Discharge | Death | Death | Discharge | Discharge | Death |

Highlighted in red are the patients with sepsis by other causes than Gram-negative bacteria for whom the model was not able to describe the correct clinical outcome.

The mean relative abundance values for *tlr4* mRNA are presented in Supplemental Table 2.

**Additional file 2: Table S2.** qPCR data from sepsis patients

| Patient # | 1 | 2 | 3 | 4 | 5 | 6 | 7 | 8 | 9 | 10 |
| --- | --- | --- | --- | --- | --- | --- | --- | --- | --- | --- |
| Day 0 | 1.0  [10 AM] | 1.0  [11.25 AM] | 1.0  [11 AM] | 1.0  [11.30 AM] | 1.0  [10 AM] | 1.0  [4 PM] | 1.0  [10 AM] | 1.0  [12 AM] | 1.0  [4 AM] | 1.0  [11 PM] |
| Day 0.5 |  |  |  | 0.6  [11.30 PM] |  | 1.97  [4 AM] | 4.3  [10 PM] | 0.56  [12 PM] | 1.97  [4 PM] |  |
| Day 1 | 0.3  [10 AM] | 0.2  [11.30 AM] | 2.5  [11 AM] |  | 0.89  [10 AM] |  |  |  |  | 1.63  [11 AM] |
| Outcome | Survival | Death | Death | Survival | Survival | Death | Death | Survival | Survival | Death |

Day 0 refers to admission to ICU and diagnosis of sepsis. Subsequent sampling was performed after 12 hours (day 0.5) or 24 hours (day 1). qPCR values expressed as arbitrary units, *tlr4* in the first line. In brackets, times of collections are indicated. qPCR data obtained from [16].

**Additional** reference:

[S1] Stan RS, Bonin C, Porto R, Soriano F, Camargo MM. **Higher levels of grp78 and lower levels of tlr4 anticipate a positive evolution of sepsis and patient survival**

BioRxiv 133264; doi: https://doi.org/10.1101/133264.
